# Diabetic kidney disease as an independent predictor of long-term adverse outcomes in patients with coronary artery disease and diabetic mellitus

**DOI:** 10.3389/fcvm.2024.1348263

**Published:** 2024-03-14

**Authors:** Zhiyu Liu, Rui Jiang, Ruochen Xu, Yunzhe Wang, Yan Lv, Chang Su, Fengyi Yu, Zhen Qin, JunNan Tang, JinYing Zhang

**Affiliations:** ^1^ Department of Cardiology, The First Affiliated Hospital of Zhengzhou University, Zhengzhou, Henan, China; ^2^ Henan Province Key Laboratory of Cardiac Injury and Repair, Zhengzhou, Henan, China; ^3^ Henan Province Clinical Research Center for Cardiovascular Diseases, Zhengzhou, Henan, China; ^4^ West China Hospital of Sichuan University, Chengdu, Sichuan, China; ^5^ Department of Medical Genetics & Cell Biology, School of Basic Medical Sciences, Zhengzhou University, Zhengzhou, China

**Keywords:** diabetic kidney disease, coronary artery disease, percutaneous coronary, major adverse cardiac and stroke events, follow-up

## Abstract

**Background:**

Diabetic kidney disease (DKD) had been proposed as a contributor in the pathogenesis of coronary artery disease (CAD). However, the relationship of DKD and the long-term adverse outcomes in patients with CAD after percutaneous coronary intervention (PCI) was still undiscovered.

**Methods:**

Approximately 892 patients with CAD enrolled from January 2012 to December 2016. The patients were divided into two groups, the DKD group (*n* = 341) and the None DKD group (*n* = 551). The primary outcome was major adverse cardiac events (MACE) after PCI. The average follow-up time was 1,897 ± 1,276 days.

**Results:**

Baseline data showed that some factors were significantly different between the two groups, including age, body mass index, gender (female), hypertension, smoking, stroke history, heart failure, duration of diabetic mellitus (DM), low-density lipoprotein cholesterol, urinary protein/creatinine ratio, serum creatinine, hemoglobin, platelet, antiplatelet, beta blocker, statin, antihypertensive drugs, and insulin (all *p* < 0.005). There were significant differences between the two groups in MACE, 40.3% vs. 52.2% (*p* = 0.001), and in cardiovascular death events and all-cause death events (5.6% vs. 20.5%, *p* < 0.001 and 4.4% vs. 13.5%, *p* < 0.001, respectively). In the DKD group, the risk of MACE was elevated to 141.9% [hazard ratio (HR) = 1.419, 95% confidence interval (CI): 1.164–1.730, *p* = 0.001] in the Cox univariable regression analyses; after adjusting co-variables, the Cox multivariable regression analyses demonstrated that DKD was an independent predictor for MACE (HR = 1.291, 95% CI: 1.027–1.624, *p* = 0.029) in patients with CAD after PCI, as well as in cardiovascular death events (HR = 2.148, 95% CI: 1.292–3.572, *p* = 0.003) and all-cause death events (HR = 2.229, 95% CI: 1.325–3.749, *p* = 0.003).

**Conclusion:**

This study suggests that DKD is an independent and novel predictor of long-term adverse outcomes in patients with CAD and DM who underwent PCI.

## 1 Introduction

Diabetic mellitus (DM) is the most common risk factor for coronary artery disease (CAD), which is the leading cause of death in the world (1). Percutaneous coronary intervention (PCI) is an effective treatment for CAD (2). However, there are still more than 30% of patients with DM after initial PCI who may experience major adverse cardiac and stroke events (MACE) during out-of-hospital treatment, including cardiovascular death, cardiac insufficiency, stroke, recurrent acute myocardial infarction, and other serious complications (3).

Relevant studies have shown that patients with diabetic kidney disease (DKD) have a higher risk of developing CAD. Among the hospitalized patients with CAD, the incidence of adverse events is higher in those with renal disease (4). In studies over the recent years, a large number of new test markers have been reported to predict the clinical prognosis of patients with CAD and type 2 DM. For example, the cystatin C or C-reactive protein (CRP) is a better indicator of the risk of CAD (5). However, there were few studies that focused on the prognosis of patients with CAD combined with DKD. Thus, we enrolled patients with DM and CAD after PCI and performed a long-term retrospective follow-up to excavate the relationship between the DKD and clinical outcomes. The Cox regressions were used as the main prediction model for data calculation.

## 2 Method

### 2.1 Study design and population

A total number of 892 patients with CAD complicated with DM were enrolled in this retrospective cohort study, and the data were collected from hospitalized inpatients at the First Affiliated Hospital of Zhengzhou University between January 2012 and December 2018. The inclusion criteria were patients with CAD including unstable angina pectoris, non-ST-elevated myocardial infarction, and ST-elevated myocardial infarction who had undergone coronary angiography (CAG) showing stenosis ≥80% and received stent implantation via PCI. According to the latest DM guidelines, a patient with DKD is defined as someone with chronic kidney disease caused by diabetes, with an estimated glomerular filtration rate (eGFR) <60 ml/(min/1.73 m^2^) and lasting for more than 3 months (6). Patients were excluded if they: (i) were lost to follow-up; or (ii) were with critical complications such as severe rheumatic heart disease, congenital heart disease, valvular heart disease, cardiomyopathy, acute infections, malignancies, hematological disease, and/or serious liver dysfunction. This study complied with the Declaration of Helsinki and the research protocol was approved by the Ethics Committee of the First Afﬁliated Hospital of Zhengzhou University (Approval number 2021-KY-0719). The details of the design and procedure are registered at http://www.chictr.org.cn (registration number: ChiCTR2200055450).

### 2.2 Demographic and clinical characteristics collection

We collected and documented the demographic data, contemporary comorbidities, and biochemical laboratory parameters for all patients, including: (i) age, body mass index (BMI), and gender (female); (ii) previously confirmed hypertension (HTN), smoking status, previous stroke, and heart failure history record; (iii) detections of high-density lipoprotein (HDL) cholesterol and low-density lipoprotein (LDL) cholesterol, urinary protein/creatinine ratio (UACR), blood serum creatinine (Scr), CRP, white blood cell (WBC), hemoglobin, and blood platelets (PLT); and (iv) management plan including antiplatelet, beta blocker, statin, hypoglycemic agent, antihypertensive drugs, and calcium channel blocker. Among these 24 baseline characteristics in Table 1 were regarded as the classic cardiovascular risk factors.

**Table 1:** Baseline characteristics between the DKD group and the None DKD group.

Variables	DKD group (341)	None DKD group (551)	*χ*^2^ or *t* or *Z*	*p*-value
Age, years	63.99 ± 10.89	54.91 ± 10.65	12.256	**<0** **.** **001**
BMI, kg m^−2^	24.27 ± 6.05	23.36 ± 6.21	−2.025	**0**.**043**
Female, *n* (%)	109 (32.0)	122 (22.1)	10.592	**0**.**001**
HTN, *n* (%)	214 (62.8)	300 (54.4)	5.957	**0**.**015**
Smoking status, *n* (%)	120 (35.2)	258 (46.8)	11.674	**0**.**001**
Stroke history, *n* (%)	69 (20.2)	58 (10.5)	16.259	**<0** **.** **001**
Heart failure, *n* (%)	228 (69.7)	294 (55.4)	17.510	**<0** **.** **001**
HbA1c, mg dl^−1^	7.53 ± 1.75	7.43 ± 1.76	0.756	0.45
Duration of DM, years	10.0 (8.0–18.0)	8.5 (8.0–11.0)	−22.773	**0**.**006**
HDL, mmol L^−1^	1.01 ± 0.29	1.03 ± 2.78	−0.791	0.429
LDL, mmol L^−1^	2.38 ± 0.93	2.53 ± 0.89	−2.333	**0**.**020**
UACR, mg g	703.97 (419.68–1,074.46)	550.78 (416.56–861.71)	2.820	**0**.**005**
Creatinine, μmol L^−1^	88.00 (77.00–101.30)	76.0 (56.00–72.00)	17.947	**<0** **.** **001**
CRP, mmol L^−1^	4.55 (1.16–17.49)	3.58 (1.02–12.45)	1.632	0.103
WBC, 10^9^ L^−1^	9.14 ± 4.24	9.50 ± 3.53	−1.299	0.194
Hemoglobin, g L^−1^	129.30 ± 24.30	136.77 ± 20.07	−4.745	**<0** **.** **001**
PLT, 10^9^ L^−1^	207.16 ± 61.04	223.28 ± 65.01	−3.493	**0**.**001**
Antiplatelet, *n* (%)	237 (72.0)	501 (91.6)	59.187	**<0** **.** **001**
Beta blocker, *n* (%)	183 (55.6)	370 (67.9)	13.284	**<0** **.** **001**
Statin, *n* (%)	261 (79.3)	509 (93.2)	37.519	**<0** **.** **001**
Calcium channel blocker, *n* (%)	28 (8.5)	40 (7.3)	0.429	0.513
Antihypertensive drugs, *n* (%)	163 (50.2)	350 (64.2)	17.358	**<0** **.** **001**
Hypoglycemic agent, *n* (%)	137 (43.2)	241 (47.2)	1.227	0.268
Insulin	138 (44.1)	152 (30.4)	15.721	**<0** **.** **001**

Factors with significant differences within the two groups are indicated in bold when *p* values < 0.05.

HTN was confirmed as an average systolic blood pressure of at least 140 mm Hg or an average diastolic blood pressure of at least 90 mm Hg or self-reported using of one or more varieties of antihypertensive medication in the past 14 days. Complying with the American Heart Association/American Diabetes Association (AHA/ADA) Scientific Statement (7), DM diagnostic criteria were positive in patients with glycated hemoglobin (A_1c_) ≥6.5% (48 mmol/mol) or fasting plasma glucose ≥126 mg/dl (7.0 mmol/L) or 2-h glucose ≥200 mg/dl (11.1 mmol/L) or in patients with random glucose ≥200 mg/dl (11.1 mmol/L) who showed classic symptoms of DM.

### 2.3 Endpoints

The primary endpoint was the MACE, including cardiovascular death events, all-cause death, hospitalization for acute cardiac insufficiency, recurrent myocardial infarction undergone PCI (re-AMI), readmission with chest pain, and stroke.

Recurrent myocardial infarction was diagnosed as a new electrocardiogram Q wave, and the plasma detection of creatine kinase MB and/or troponin I/T increased to more than threefold of the upper limit of the normal range within 48 h after the procedure (8). Stroke was deﬁned as sudden onset of syncope, aphasia, and decreased level of consciousness, due to hemorrhage, embolism, or aneurysm rupture, that persisting for >24 h (9). All event incidences were verified by an event adjudication committee that was blinded to the group of participants.

### 2.4 Follow-up period

**Figure 1:**
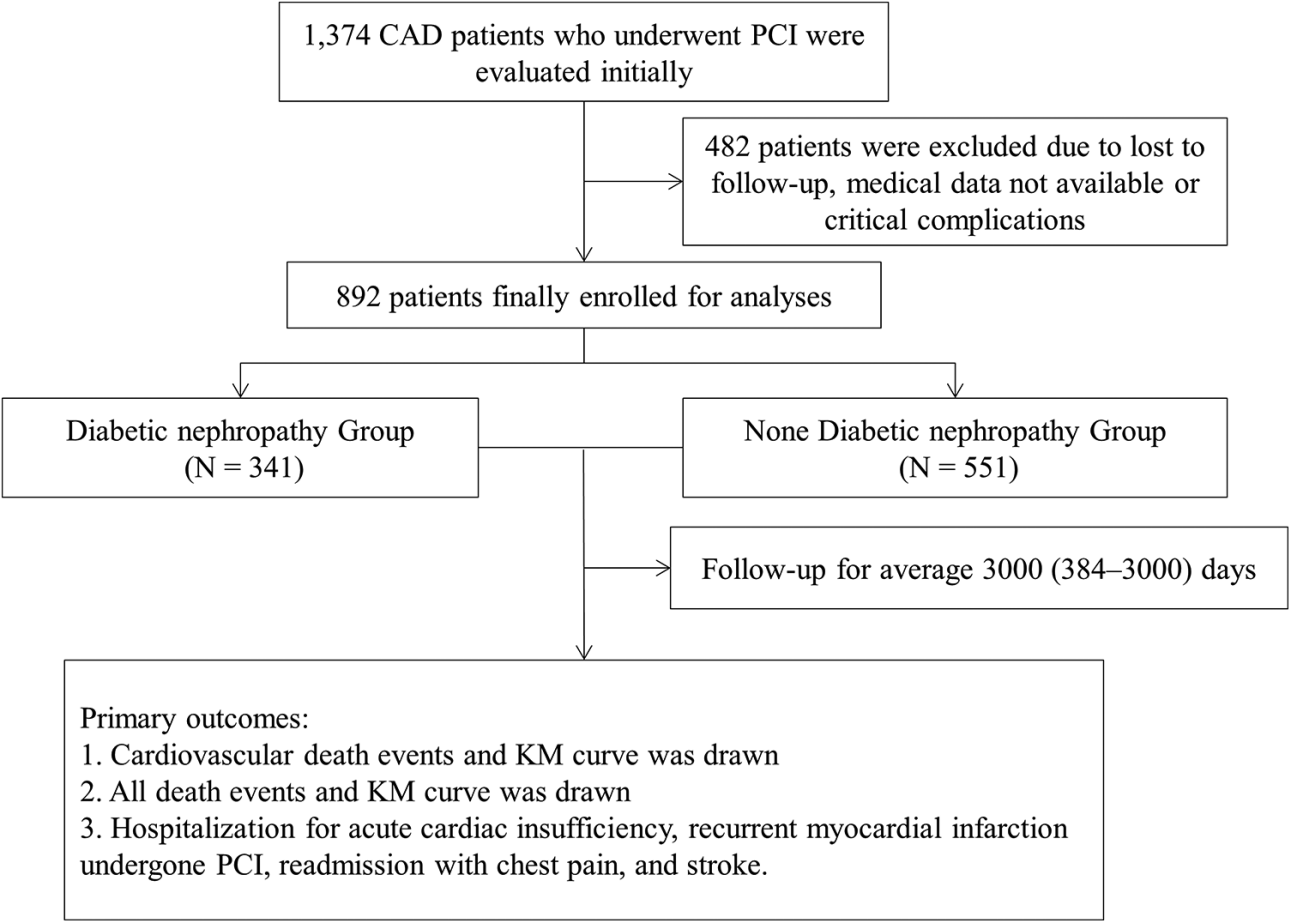
The flowchart of the patients’ enrollment.

In this research, the median follow-up time was 3,000.0 days (384.0–3,000.0). The follow-up visits were performed by physicians in out-patient department offices or by telephone contacts, as necessary. The endpoint events were assessed at each follow-up visit. Flow chart of the study is shown in Figure 1.

### 2.5 Statistical analyses

All analyses were performed using the SPSS 24.0 for Windows statistical software. The continuous variables were tested for normal distribution using the Mann–Whitney *U* test, and presented as mean ± standard deviation (SD). They were compared using two-tailed unpaired *t* tests; non-normal data were expressed as median (lower quartile, upper quartile). The categorical variables were demonstrated as numbers and percentages and compared using the two-tailed *χ*^2^ test, and presented as numbers (percentage). The Kaplan–Meier curves were framed for cumulative incidence rates of long-term outcomes and the log-rank test was used to compare variables between groups. Cox univariable regression analyses were used to collect the covariates with the DKD group, which were enrolled into the Cox multivariable regression when *p* < 0.1 (bold font in Tables 3–5); Cox multivariable regression analyses were performed to estimate the predictive value of the DKD group for outcomes, using backward stepwise regression. Hazard ratios (HRs) and 95% conﬁdence intervals (CIs) were calculated, and *p* < 0.05 was considered statistically signiﬁcant. Collinearity diagnostic test was used to certify whether there is a high correlation between independent variables in the Cox multivariable regression.

**Table 3:** Analyses of Cox univariable model for MACE events.

Variables	B	SE	Wald	Hazard ratio	95% confidence interval	*p*-value
DKD groups	0.350	0.101	11.965	1.419	1.164–1.730	**0**.**001**
Age	0.006	0.004	1.715	1.006	0.997–1.014	0.190
Female	−0.024	0.115	0.045	0.976	0.780–1.222	0.833
HTN	−0.053	0.102	0.267	0.949	0.778–1.158	0.605
Smoking status	0.181	0.101	3.209	1.198	0.983–1.460	**0**.**073**
Stroke history	0.123	0.138	0.787	1.131	0.862–1.482	0.375
Heart failure	0.105	0.104	1.020	1.110	0.906–1.361	0.313
HbA1c	0.076	0.028	7.539	1.079	1.022–1.139	**0**.**006**
Duration of DM	0.017	0.008	5.111	1.017	1.002–1.032	**0**.**024**
HDL	−0.198	0.186	1.133	0.820	0.569–1.182	0.287
LDL	−0.100	0.058	2.944	0.905	0.807–1.014	**0**.**086**
Creatinine	0.000	0.001	0.465	1.000	0.999–1.002	0.495
CRP	0.000	0.000	0.274	1.000	1.000–1.000	0.600
WBC	0.002	0.014	0.020	1.002	0.974–1.031	0.889
Hemoglobin	−0.005	0.002	4.508	0.995	0.991–1.000	**0**.**034**
PLT	−0.002	0.001	3.925	0.998	0.997–1.000	**0**.**048**
Antiplatelet	−0.271	0.132	4.196	0.763	0.588–0.988	**0**.**041**
Beta blocker	−0.174	0.105	2.738	0.840	0.684–1.033	0.098
Statin	−0.407	0.143	8.122	0.666	0.503–0.881	**0**.**004**
Calcium channel blocker	−0.229	0.207	1.224	0.795	0.530–1.193	0.269
Antihypertensive drugs	−0.121	0.104	1.350	0.886	0.723–1.087	0.245
Hypoglycemic agent	−0.040	0.101	0.159	0.960	0.787–1.172	0.690
Insulin	0.138	0.104	1.750	1.148	0.936–1.408	0.186

**Table 4:** Analyses of Cox univariable model for cardiovascular death events.

Variables	B	SE	Wald	Hazard ratio	95% confidence interval	*p*-value
DKD groups	1.374	0.216	40.464	3.951	2.587–6.033	**<0**.**001**
Age	0.076	0.010	61.679	1.079	1.059–1.100	**<0**.**001**
Female	0.326	0.212	2.366	1.386	0.914–2.101	0.124
HTN	−0.178	0.200	0.791	0.837	0.566–1.239	0.374
Smoking status	−0.301	0.208	2.102	0.740	0.492–1.112	0.147
Stroke history	0.328	0.255	1.654	1.388	0.842–2.286	0.198
Heart failure	1.029	0.243	17.937	2.797	1.738–4.502	**<0**.**001**
HbA1c	0.084	0.054	2.365	1.087	0.977–1.210	0.124
Duration of DM	0.032	0.014	5.531	1.033	1.005–1.061	**0**.**019**
HDL	0.509	0.340	2.242	1.663	0.855–3.238	0.134
LDL	−0.128	0.118	1.168	0.880	0.699–1.109	0.280
Creatinine	0.002	0.001	12.253	1.002	1.001–1.003	**<0**.**001**
CRP	0.000	0.000	0.049	1.000	1.000–1.000	0.826
WBC	0.068	0.026	6.957	1.070	1.018–1.126	**0**.**008**
Hemoglobin	−0.007	0.004	2.596	0.993	0.985–1.001	0.107
PLT	−0.004	0.002	5.537	0.996	0.992–0.999	**0**.**019**
Antiplatelet	−1.468	0.219	44.915	0.230	0.150–0.354	**<0**.**001**
Beta blocker	−1.108	0.223	24.601	0.330	0.213–0.512	**<0**.**001**
Statin	−1.887	0.219	74.220	0.152	0.099–0.233	**<0**.**001**
Calcium channel blocker	−0.400	0.462	0.753	0.670	0.271–1.655	0.386
Antihypertensive drugs	−1.190	0.235	25.571	0.304	0.192–0.482	**<0**.**001**
Hypoglycemic agent	−1.182	0.246	23.133	0.307	0.190–0.497	**<0**.**001**
Insulin	0.058	0.211	0.075	1.059	0.701–1.601	0.785

**Table 5:** Analyses of Cox univariable model for all-cause death events.

Variables	B	SE	Wald	Hazard ratio	95% confidence interval	*p*-value
DKD groups	1.196	0.252	22.562	3.308	2.019–5.420	**<0**.**001**
Age	0.074	0.012	41.023	1.077	1.053–1.102	**<0**.**001**
Female	0.188	0.261	0.517	1.206	0.723–2.012	0.472
HTN	−0.402	0.239	2.825	0.669	0.419–1.069	**0**.**093**
Smoking status	−0.478	0.257	3.450	0.620	0.374–1.027	**0**.**063**
Stroke history	0.229	0.317	0.523	1.258	0.676–2.342	0.470
Heart failure	0.953	0.292	10.672	2.594	1.464–4.597	**0**.**001**
HbA1c	−0.093	0.084	1.239	0.911	0.773–1.074	0.266
Duration of DM	−0.003	0.020	0.017	0.997	0.959–1.037	0.897
HDL	0.400	0.427	0.877	1.491	0.646–3.441	0.349
LDL	−0.165	0.145	1.295	0.848	0.638–1.127	0.255
Creatinine	0.002	0.001	6.861	1.002	1.001–1.004	**0**.**009**
CRP	0.000	0.000	0.021	1.000	1.000–1.000	0.884
WBC	0.103	0.026	15.761	1.109	1.054–1.167	**<0**.**001**
Hemoglobin	−0.006	0.005	1.507	0.994	0.984–1.004	0.220
PLT	−0.005	0.002	5.880	0.995	0.990–0.999	**0**.**015**
Antiplatelet	−0.519	0.318	2.658	0.595	0.319–1.111	0.103
Beta blocker	−0.498	0.273	3.337	0.608	0.356–1.037	**0**.**068**
Statin	−0.872	0.318	7.491	0.418	0.224–0.781	**0**.**006**
Calcium channel blocker	−0.389	0.594	0.429	0.678	0.211–2.171	0.512
Antihypertensive drugs	−0.652	0.274	5.661	0.521	0.304–0.891	**0**.**017**
Hypoglycemic agent	−1.214	0.299	16.431	0.297	0.165–0.534	**<0**.**001**
Insulin	−0.771	0.300	6.624	0.462	0.257–0.832	**0**.**010**

## 3 Results

### 3.1 Baseline demographic and clinical characteristics

As presented in Table 1, several variables showed signiﬁcant differences between these two groups, including age (63.99 ± 10.89 vs. 54.91 ± 10.65, *p *< 0.001), BMI (24.27 ± 6.05 vs. 23.36 ± 6.21, *p *= 0.043), gender (female, 32.0% vs. 22.1%, *p *= 0.001), HTN (62.8% vs. 54.4%, *p *= 0.015), smoking status (35.2% vs. 46.8%, *p *= 0.001), stroke history (20.2% vs. 10.5%, *p *< 0.001), heart failure (69.7% vs. 55.4%, *p *< 0.001), duration of DM [10.0 (8.0–18.0) vs. 8.5 (8.0–11.0), *p *< 0.001], LDL (2.38 ± 0.93 vs. 2.53 ± 0.89, *p *= 0.020), UACR [703.97 (419.68–1,074.46) vs. 550.78 (416.56–861.71), *p *= 0.005], serum creatinine [88.00 (77.00–101.30) vs. 76.0 (56.00–72.00), *p* < 0.001], hemoglobin (129.30 ± 24.30 vs. 136.77 ± 20.07, *p *< 0.001), platelet (207.16 ± 61.04 vs. 223.28 ± 65.01, *p *< 0.001), antiplatelet (72.0% vs. 91.6%, *p *< 0.001), beta blocker (55.6% vs. 67.9%, *p *< 0.001), statin (79.3% vs. 93.2%, *p *< 0.001), antihypertensive drugs (50.2% vs. 64.2%, *p *< 0.001), and insulin 44.1% vs. 30.4%, *p *< 0.001. However, signiﬁcant differences between the two groups were not found in HbA1c, HDL, CRP, WBC, hypoglycemic agent, and calcium channel blocker comparisons (all *p* > 0.05).

### 3.2 Clinical outcomes

As demonstrated in Table 2, the MACE incidents were less in the None DKD group vs. the DKD group (40.3% vs. 52.2%, *p* = 0.001). For the cardiovascular death events and all-cause death events, we found significant differences between the two groups (5.6% vs. 20.5%, *p *< 0.001; and 4.4% vs. 13.5%, *p *< 0.001). Signiﬁcant differences between the two groups were not found in hospitalization for acute cardiac insufficiency, recurrent myocardial infarction undergone PCI, readmission with chest pain, and stroke.

**Table 2:** Outcomes comparison between the two groups.

Outcomes	DKD group	None DKD group	*χ*^2^ or *t*	*p*-value
MACE	178 (52.2)	222 (40.3)	12.078	**0**.**001**
Cardiovascular death events^a^	70 (20.5)	31 (5.6)	46.585	**<0**.**001**
All-cause death^a^	46 (13.5)	24 (4.4)	24.301	**<0**.**001**
Hospitalization for acute cardiac insufficiency	47 (13.8)	52 (9.4)	5.7	0.058
re-AMI	48 (14.1)	70 (12.7)	0.345	0.557
Readmission with chest pain	106 (31.1)	163 (29.6)	0.226	0.635
Stroke	4 (1.3)	6 (1.2)	0.007	0.933

Factors with significant differences within the two groups are indicated in bold when *p* values < 0.05.

^a^ Cardiovascular death events and hospitalization for acute cardiac insufficiency Kaplan–Meier curves were showed in Figures 3, 4.

**Figure 2:**
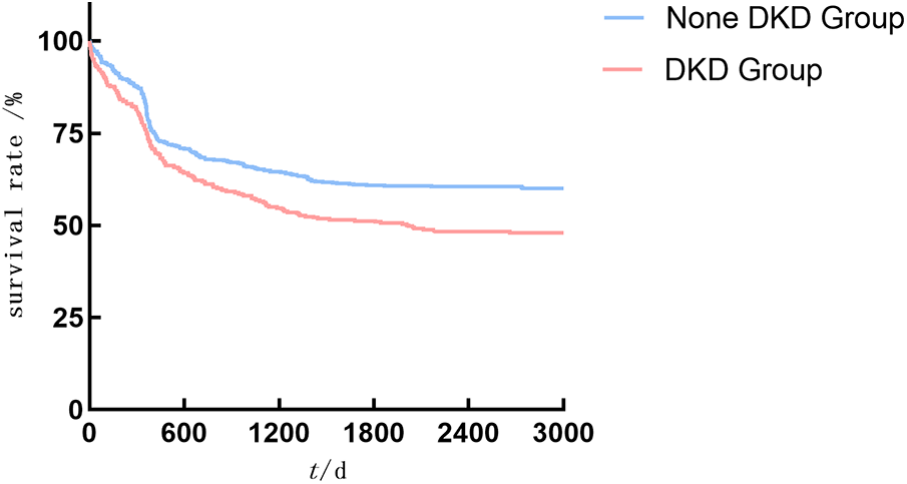
The DKD group vs. the None DKD group's Kaplan–Meier curves for MACE.

**Figure 3:**
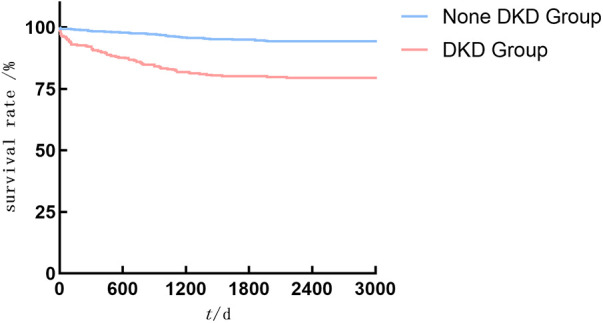
The DKD group vs. the None DKD group's Kaplan–Meier curves for cardiovascular death events.

**Figure 4:**
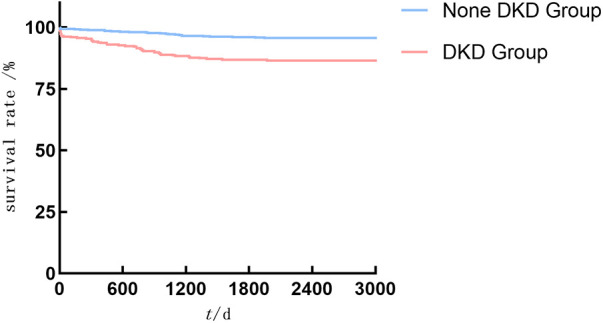
The DKD group vs. the None DKD group's Kaplan–Meier curves for all-cause death events.

As shown in Figures 2–4, the DKD group vs. the None DKD group's Kaplan–Meier curves were framed for cumulative incidence rates of long-term outcomes and the log-rank *p* < 0.001 between groups.

### 3.3 Univariable Cox regression

As presented in Table 3, all baseline characteristics were calculated in the MACE by univariable Cox regression analyses. We found that belonging to the DKD group, smoking status, HbA1c, duration of DM, hemoglobin, PLT, antiplatelet, and statin were predictors. Belonging to the DKD group is a risk factor and the HR of MACE was 1.419 (95% CI: 1.164–1.730, *p* = 0.001). Smoking status, HbA1c, duration of DM were also risk factors (HR = 1.198, 1.079, and 1.017; *p = *0.073, 0.006, and 0.024), and higher hemoglobin, higher PLT, antiplatelet medication group, and statin medication group were protective factors (HR = 0.995, 0.998, 0.763, and 0.666; *p = *0.034, 0.048, 0.041, and 0.004).

As presented in Table 4, all baseline characteristics were calculated in cardiovascular death events by univariable Cox regression analyses, and as a result, belonging to the DKD group, age, heart failure, duration of DM, creatinine, WBC, PLT, antiplatelet, beta blocker, statin, antihypertensive drugs and hypoglycemic agent were predictors. The DKD group is a risk factor and the HR of MACE was 3.951 (95% CI: 2.587–6.033, *p* < 0.001). Age, heart failure, duration of DM, creatinine, WBC were also risk factors (HR, all >1.0; *p <* 0.05), and PLT, antiplatelet, beta blocker, statin, antihypertensive drugs, and hypoglycemic agent were protective factors (all HR <1.0; *p <* 0.05).

As presented in Table 5, all baseline characteristics were calculated in all-cause death events by univariable Cox regression analyses, and as a result, belonging to the DKD group, age, HTN, smoking status, heart failure, creatinine, WBC, PLT, beta blocker, statin, antihypertensive drugs, hypoglycemic agent, insulin were predictors. The DKD group is a risk factor and the HR of MACE was 3.308 (95% CI: 2.019–5.420, *p* < 0.001). HTN, smoking status, heart failure, creatinine, WBC were also risk factors (HR all >1.0; *p <* 0.05), and PLT, beta blocker, statin, antihypertensive drugs, hypoglycemic agent, and insulin were protective factors (HR all <1.0; *p <* 0.05).

### 3.4 Multivariable Cox regression

In Table 6, after adjusting for univariable factors from Table 3 (Smoking status, HbA1c ≥ 9.0 mmol L^−1^, LDL ≥ 1.8 mmol L^−1^), compared with the patients in the None DKD group, in the DKD group the risk for MACE was elevated to 129.1% (HR = 1.291, 95% CI: 1.027–1.624, *p* = 0.029) in the multivariable model, and the DKD group was an independent predictor to the MACE in patients with CAD accompanied with DM after PCI. These four factors were not collinear [Variance Inflation Factor (VIF) < 5.0].

**Table 6:** Analyses of Cox multivariable model for MACE event.

Variables	B	SE	Wald	Hazard ratio	95% confidence interval	*p*-value	VIF
DKD groups	0.256	0.117	4.776	1.291	1.027–1.624	0.029	1.028
Smoking status	0.227	0.114	3.938	1.255	1.003–1.570	0.047	1.025
HbA1c ≥ 9.0 mmol L^−1^	0.339	0.141	5.788	1.403	1.065–1.849	0.016	1.015
LDL ≥ 1.8 mmol L^−1^	−0.299	0.128	5.477	0.742	0.577–0.953	0.019	1.013

In Table 7, after adjusting for univariable factors from Table 4 (age ≥60 years, heart failure, statin, hypoglycemic agent), compared with the patients in the None DKD group, in the DKD group the risk for cardiovascular death events were elevated to 214.8% (HR = 2.148, 95% CI: 1.292–3.572, *p* = 0.003) in the multivariable model, and the DKD group was an independent predictor to the MACE in patients with CAD accompanied with DM after PCI. These five factors were not collinear (VIF < 5.0).

**Table 7:** Analyses of Cox multivariable model for cardiovascular death events.

Variables	B	SE	Wald	Hazard ratio	95% confidence interval	*p*-value	VIF
DKD groups	0.764	0.259	8.678	2.148	1.292–3.572	0.003	1.168
Age ≥60 years	1.248	0.313	15.859	3.482	1.884–6.435	<0.001	1.160
Heart failure	0.583	0.262	4.949	1.791	1.072–2.992	0.026	1.033
Statin	−0.849	0.261	10.560	0.428	0.256–.0,714	0.001	1.167
Hypoglycemic agent	−1.068	0.315	11.501	0.344	0.185–0.637	0.001	1.102

In Table 8, after adjusting for univariable factors from Table 5 (age ≥60 years, hypoglycemic agent), compared with the patients in the None DKD group, in the DKD group the risk for MACE was elevated to 222.9% (HR = 2.229, 95% CI: 1.325–3.749, *p* = 0.003) in the multivariable model, and the DKD group was an independent predictor to the all-cause death event in the patients with CAD accompanied with DM after PCI. These four factors were not collinear (VIF < 5.0).

**Table 8:** Analyses of Cox multivariable model for all-cause death events.

Variables	B	SE	Wald	Hazard ratio	95% confidence interval	*p*-value	VIF
DKD groups	0.801	0.265	9.122	2.229	1.325–3.749	0.003	1.125
Age ≥60 years	1.170	0.306	14.593	3.224	1.768–5.877	<0.001	1.126
Hypoglycemic agent	−1.270	0.300	17.958	0.281	0.156–0.505	<0.001	1.005

## 4 Discussion

The prevalence of type 2 DM is increasing worldwide because of the increasing number of obese patients (10), DKD affects about 25% of patients with type 2 diabetes and is one of the leading cause of death in the world in high-income countries (11). In addition, patients with DKD have a very high cardiovascular risk, comparable with patients without DKD (12). In these patients, treatment with statins, angiotension-converting enzyme (ACE) inhibitors, or angiotensin-receptor blockers can delay the progression of kidney disease and reduce cardiovascular morbidity, whereas glucose-lowering therapy can prevent and delay the progression of DKD (13).

The main cause of death in patients with CKD and DM is cardiovascular death, which is caused by events such as stroke, sudden cardiac arrest, myocardial infarction, and other fatal complications. Numerous studies have shown that accelerated aging of blood vessels, valves, and the heart, characterized by soft tissue calcification, vascular wall degeneration, and myocardial fibrosis, is associated with mortality in patients with DM and CKD (14–16). Therefore, identification and management of risk factors for DKD, as well as timely diagnosis and treatment of the disease, are essential.

Extensive endothelial dysfunction, characterized by increased vascular permeability and inflammation (17), is a pathogenic mechanism underlying multiple microvascular and macrovascular complications of diabetes and is associated with cardiovascular mortality (17). In this study, we had found the DKD group HR indicates a 41.9% increase in the risk of MACE (HR = 1.419, 95% CI: 1.164–1.730, *p* = 0.001) in univariable Cox regression. After adjusting the co-variables, including smoking status, HbA1c ≥ 9.0 mmol L^−1^, LDL ≥ 1.8 mmol L^−1^, and the DKD group, the risk for MACE was elevated to 129.1% (HR = 1.376, 95% CI: 1.027–1.624, *p* = 0.029), thus the DKD group was an independent predictor to the risk of MACE event.

Consistent with these findings, for broadly defined renal failure [RF, refers to eGFR < 60 ml/(min/1.73 m^2^)] in patients with DM, Liosis et al. used a *post hoc* study to examine in-hospital term mortality in patients with acute coronary syndrome, and found that patients with both diabetes and RF had the highest mortality compared with other subgroups of patients (18). Compared with the no-DM and the no-RF subgroup, the odds ratios of DM without RF, DM with RF, RF without DM subgroups were: 1.62, 3.0, and 2.13, respectively. Watanabe et al. evaluated the RF in patients with left main distal bifurcation, and found that adverse outcomes were significantly higher in the severe RF group than that in the other groups (adjusted HR, 3.6) (19). They suggested that vigilance for the complications of DKD should be maintained when developing treatment strategies for patients after PCI, or other surgical procedures for coronary artery treatment, such as coronary artery bypass grafting (CABG) (20). Moreover, the increased risk is not only for CAD, but also for peripheral vascular diseases such as diabetic retinopathy or stroke (21).

In DKD, significant inflammation can be observed both in the beginning and ongoing stages of renal injury (22). Both systemic and local renal inflammatory responses were upregulated in DKD. Sapa et al. found that an inflammatory marker of renal filtration, asymmetric dimethylarginine (ADMA), had demonstrated a direct connection between low renal uremic solute clearance and the deterioration of cardiovascular death (23). In the diabetic state, high glucose and oxidative stress simultaneously induce the activation of the known inflammatory signaling molecule NF-κB (24), which triggers activation of the downstream effector iNOS, a promoter of oxidative stress and inflammation, leading to extensive nitrotyrosine in proteins (25). Sun et al. found that pharmacological inhibition of NF-κB signaling can prevent DKD (26).

There were limitations in this study, it was a single-center study with the limitation of regional selection, and the retrospective study caused limited data collection bias, which needs to be improved by future prospective studies. Therefore, further a prospective multi-center cohort study with a continuous estimation would improve the evidence level of evidence-based medicine. In conclusion, in this study we had found that DKD is an independent and novel predictor of long-term adverse outcomes in patients with CAD accompanied with DM who underwent PCI.

## Data Availability

The raw data supporting the conclusions of this article will be made available by the authors, without undue reservation.

## References

[1] Yahagi K Kolodgie FD Lutter C Mori H Romero ME Finn AV Pathology of human coronary and carotid artery atherosclerosis and vascular calcification in diabetes mellitus. Arterioscler Thromb Vasc Biol. (2017) 37(2):191–204. 10.1161/atvbaha.116.306256 27908890 PMC5269516

[2] Denktas AE Paniagua D Jneid H. Coronary physiology assessment for the diagnosis and treatment of stable ischemic heart disease. Curr Atheroscler Rep. (2016) 18(10):62. 10.1007/s11883-016-0613-2 27686574

[3] Huang Z Chan TM Dong W. MACE prediction of acute coronary syndrome via boosted resampling classification using electronic medical records. J Biomed Inform. (2017) 66:161–70. 10.1016/j.jbi.2017.01.001 28065840

[4] Ferrannini G Manca ML Magnoni M Andreotti F Andreini D Latini R Coronary artery disease and type 2 diabetes: a proteomic study. Diabetes Care. (2020) 43(4):843–51. 10.2337/dc19-1902 31988066

[5] Rifai N Ridker PM. High-sensitivity C-reactive protein: a novel and promising marker of coronary heart disease. Clin Chem. (2001) 47(3):403–11. 10.1093/clinchem/47.3.403 11238289

[6] American Diabetes Association. 2. Classification and diagnosis of diabetes: standards of medical care in diabetes—2020. Diabetes Care. (2020) 43(Suppl 1):S14–31. 10.2337/dc20-S002 31862745

[7] American Diabetes Association. Classification and diagnosis of diabetes: standards of medical care in diabetes—2018. Diabetes Care. (2018) 41(Suppl 1):S13–27. 10.2337/dc18-S002 29222373

[8] Li SJ Ge Z Kan J Zhang JJ Ye F Kwan TW Cutoff value and long-term prediction of clinical events by FFR measured immediately after implantation of a drug-eluting stent in patients with coronary artery disease: 1- to 3-year results from the DKCRUSH VII registry study. JACC Cardiovasc Interv. (2017) 10(10):986–95. 10.1016/j.jcin.2017.02.012 28456699

[9] Sacco RL Kasner SE Broderick JP Caplan LR Connors JJ Culebras A An updated definition of stroke for the 21st century: a statement for healthcare professionals from the American Heart Association/American Stroke Association. Stroke. (2013) 44(7):2064–89. 10.1161/STR.0b013e318296aeca 23652265 PMC11078537

[10] Lovic D Piperidou A Zografou I Grassos H Pittaras A Manolis A. The growing epidemic of diabetes mellitus. Curr Vasc Pharmacol. (2020) 18(2):104–9. 10.2174/1570161117666190405165911 30961501

[11] Umanath K Lewis JB. Update on diabetic nephropathy: core curriculum 2018. Am J Kidney Dis. (2018) 71(6):884–95. 10.1053/j.ajkd.2017.10.026 29398179

[12] Yu D Wang Z Zhang X Qu B Cai Y Ma S Remnant cholesterol and cardiovascular mortality in patients with type 2 diabetes and incident diabetic nephropathy. J Clin Endocrinol Metab. (2021) 106(12):3546–54. 10.1210/clinem/dgab533 34291804

[13] Karalliedde J Winocour P Chowdhury TA De P Frankel AH Montero RM Clinical practice guidelines for management of hyperglycaemia in adults with diabetic kidney disease. Diabet Med. (2022) 39(4):e14769. 10.1111/dme.14769 35080257

[14] Hahr AJ Molitch ME. Management of diabetes mellitus in patients with CKD: core curriculum 2022. Am J Kidney Dis. (2022) 79(5):728–36. 10.1053/j.ajkd.2021.05.023 34600745

[15] Collins AJ Pitt B Reaven N Funk S Mcgaughey K Wilson D Association of serum potassium with all-cause mortality in patients with and without heart failure, chronic kidney disease, and/or diabetes. Am J Nephrol. (2017) 46(3):213–21. 10.1159/000479802 28866674 PMC5637309

[16] Deng Y Li N Wu Y Wang M Yang S Zheng Y Global, regional, and national burden of diabetes-related chronic kidney disease from 1990 to 2019. Front Endocrinol. (2021) 12:672350. 10.3389/fendo.2021.672350 PMC8281340 34276558

[17] Yang J Liu Z. Mechanistic pathogenesis of endothelial dysfunction in diabetic nephropathy and retinopathy. Front Endocrinol. (2022) 13:816400. 10.3389/fendo.2022.816400 PMC9174994 35692405

[18] Liosis S Hochadel M Darius H Behrens S Mudra H Lauer B Effect of renal insufficiency and diabetes mellitus on in-hospital mortality after acute coronary syndromes treated with primary PCI. Results from the ALKK PCI registry. Int J Cardiol. (2019) 292:43–9. 10.1016/j.ijcard.2019.04.071 31088759

[19] Watanabe Y Mitomo S Naganuma T Chieffo A Montorfano M Nakamura S Impact of chronic kidney disease in patients with diabetes mellitus after percutaneous coronary intervention for left main distal bifurcation (from the Milan and New-Tokyo (MITO) registry). Am J Cardiol. (2021) 138:33–9. 10.1016/j.amjcard.2020.10.021 33058802

[20] Bae KS Park HC Kang BS Park JW Chon NR Oh KJ Percutaneous coronary intervention versus coronary artery bypass grafting in patients with coronary artery disease and diabetic nephropathy: a single center experience. Korean J Intern Med. (2007) 22(3):139–46. 10.3904/kjim.2007.22.3.139 17939329 PMC2687692

[21] Yen FS Wei JC Shih YH Hsu CC Hwu CM. Impact of individual microvascular disease on the risks of macrovascular complications in type 2 diabetes: a nationwide population-based cohort study. Cardiovasc Diabetol. (2023) 22(1):109. 10.1186/s12933-023-01821-8 37161539 PMC10170797

[22] Pérez-Morales RE Del Pino MD Valdivielso JM Ortiz A Mora-Fernández C Navarro-González JF. Inflammation in diabetic kidney disease. Nephron. (2019) 143(1):12–6. 10.1159/000493278 30273931

[23] Sapa H Gutiérrez OM Shlipak MG Katz R Ix JH Sarnak MJ Association of uremic solutes with cardiovascular death in diabetic kidney disease. Am J Kidney Dis. (2022) 80(4):502–12.e501. 10.1053/j.ajkd.2022.02.016 35351578 PMC9554797

[24] Huang S Wang J Zhang L Tian S Wang Y Shao X Ras guanine nucleotide-releasing protein-4 promotes renal inflammatory injury in type 2 diabetes mellitus. Metab Clin Exp. (2022) 131:155177. 10.1016/j.metabol.2022.155177 35218794

[25] Khan S Jena G Tikoo K Kumar V. Valproate attenuates the proteinuria, podocyte and renal injury by facilitating autophagy and inactivation of NF-*κ*B/iNOS signaling in diabetic rat. Biochimie. (2015) 110:1–16. 10.1016/j.biochi.2014.12.015 25572918

[26] Sun HJ Xiong SP Cao X Cao L Zhu MY Wu ZY Polysulfide-mediated sulfhydration of SIRT1 prevents diabetic nephropathy by suppressing phosphorylation and acetylation of p65 NF-*κ*B and STAT3. Redox Biol. (2021) 38:101813. 10.1016/j.redox.2020.101813 33279869 PMC7718489

